# Supplementary material for: Singular states of resonant nanophotonic lattices
Source: Nanophotonics. 2023 Jan 4;12(2):263–72. doi: 10.1515/nanoph-2022-0608 (PMC11501766; doi:10.1515/nanoph-2022-0608)
Supplement: Supplementary file 1 — Supplementary Material Details [file j_nanoph-2022-0608_suppl.docx]

Supplementary Materials

Yeong Hwan Ko, Kyu Jin Lee, Fairooz Abdullah Simlan and Robert Magnusson*

Singular states of resonant nanophotonic lattices

Department of Electrical Engineering, University of Texas at Arlington, Arlington, Texas 76019, USA

^*^E-mail: [magnusson@uta.edu](mailto:magnusson@uta.edu)

1 Modelling external zero-order transmittance ($\boldsymbol{T}_{\boldsymbol{0}}^{\boldsymbol{'}}$) including substrate reflection

The measured transmittance of the fabricated devices (i.e., Si membrane encapsulated by quartz and index-matching oil) can be anticipated by modelling external zero-order transmittance ($T_{0}^{'}$) accounting for substrate reflection. As illustrated in Fig. S1, input light passes through the host medium (n=1.45) with surface loss by reflection where the transmittance is estimated by the Fresnel equations ($T_{in}$). Then, the light enters the Si (n_Si_=3.48) membrane with transmittance $T_{0}$. Thereafter, the light exits with surface transmission ($T_{in}T_{0}T_{out}$) and partially bounces to the Si membrane. In regard to this cyclical sequence, the *n*th external transmittance $T_{\left( n \right)}^{'}$ is expressed as

$T_{\left( n \right)}^{'}=T_{in}T_{0}\left\{ \left( 1-T_{out} \right)T_{0}\left( 1-T_{in} \right)T_{0} \right\}^{n}T_{out}\approx T_{in}^{2}T_{0}\left\{ \left( 1-T_{in} \right)T_{0} \right\}^{2n}$  **(1)**

Figure S1. Modelling a Si lattice accounting for boundaries of a finite host medium.

where $T_{out}\approx T_{in}$ is applied as an approximation. Finally, the total external transmittance $T_{0}^{'}$ is

$T_{0}^{'}=\sum_{n=0}^{\infty} T_{\left( n \right)}^{'}=T_{in}^{2}T_{0}\sum_{n=0}^{\infty} \left\{ \left( 1-T_{in} \right)T_{0} \right\}^{2n}=\frac{T_{in}^{2}T_{0}}{1-\left\{ \left( 1-T_{in} \right)T_{0} \right\}^{2n}}$ **(2)**

Here, $T_{0}$ is computed numerically and $T_{in}$=0.966. The $T_{0}$ and $T_{0}^{'}$ spectra are compared with the measured spectra in Fig. S2. Accounting for the host media boundaries brings the simulation results closer to the measured data.

Figure S2. Comparison of the (a) $T_{0}$ and (b) $T_{0}^{'}$ spectra. The parameter set of the fabricated device is (*Λ*=0.835 µm, *F*=0.396 and *H*=0.33 µm) and the input wave is TE polarized.

2 Fabricated device performance under fill-factor and divergence variation

To explain the broadened peaks of the singular state, we analyze $T_{0}^{'}$ spectra accounting for imperfect fabrication and the divergence of the input beam.

2.1 Fill factor variation ($\boldsymbol{F’}$)

As a main grating parameter, the *F* (fill factor) is varied by $F^{'}=F\left( 1+\Delta F \right)$ where $\Delta F$ is a deviation from the design *F*. Figure S3 shows the perturbed $T_{0}^{'}$ (thin solid line) and averaged $T_{0}^{'}$ (thick solid line) spectra at (a) $\theta=5^{o}$ and (b) ${10}^{o}$. At both angles, the aTE_0_ (asymmetric GMR) dip is broadened and degraded whereas the aTE_1_ peak is more robust. This is because the aTE_0_ mode is more sensitive to *F* than the aTE_1_ mode as can be shown by EMT modelling. At $\theta=5^{o}$, the aTE_0_ is dramatically degraded because of its narrow resonance linewidth. For $\Delta F=\pm1.5\%$ variation, based on AFM data, the averaged aTE_0_ dip is at 0.45 and 0.11 at $\theta=5^{o}$ and ${10}^{o}$, respectively.

Figure S3. Perturbed $T_{0}^{'}$ by fill-factor variation $F^{'}=F\left( 1+\Delta F \right)$ at (a) $\theta=5^{o}$ and (b) ${10}^{o}$ where $\Delta F$ is at $\pm1.5\%$. Parameter set is the same as for Fig. S2.

2.2 Angular variation ($\boldsymbol{\Delta}\boldsymbol{\theta}$)

The angle of incidence ($\theta$) is varied by $\theta+\Delta\theta$ where the $\Delta\theta$ is the deviation from the plane-wave angle $\theta$. In Figs. S4(a) and S4(b), the perturbed and averaged $T_{0}^{'}$ spectra are shown at (a) $\theta=5^{o}$ and (b) ${10}^{o}$. At both angles, the aTE_0_ dip is degraded slightly whereas the aTE_1_ peak is significantly reduced. For $\Delta\theta=\pm1^{o}$ variation, the averaged aTE_1_ peak is 0.55 and 0.79 at $\theta=5^{o}$ and ${10}^{o}$, respectively.

Figure S4. Perturbed $T_{0}^{'}$ by angular deviation in (a) $5^{o}+\Delta\theta$ and (b) ${10}^{o}+\Delta\theta$ where $\Delta\theta$ is varied as $\pm1^{o}$. Parameter set is the same as for Fig. S2.

2.3 Simultaneous variation of fill factor ($\boldsymbol{F’}$) and input angle ($\boldsymbol{\Delta}\boldsymbol{\theta}$)

Under simultaneous variation of $F^{'}$ and $\Delta\theta$, in Fig. S5, the perturbed and averaged $T_{0}^{'}$ spectra are compared with measured spectra for (a) $\theta=5^{o}$ and (b) ${10}^{o}$. At both incidence angles, the theoretical spectra approximate the measured spectra. This supports the contention that fill-factor variation across the device and angular components leading to imperfect beam collimation are main effects causing deviation between theory and experiment.

Figure S5. Experimental spectra compared with $T_{0}^{'}$ spectra computed under concomitant variation of $\Delta F=\pm1.5\%$ and $\Delta\theta=\pm1^{o}$ (a) $\theta=5^{o}$ and (b) ${10}^{o}$. Parameter set is the same as for Fig. S2.
